# Music and Anesthesia

**Published:** 1901-06-15

**Authors:** 


### MUSIC AND ANESTHESIA.

According to the *Daily Telegraph* musical anesthesia has been taken up by a well-known physician, who expounded it before the Paris Academy of Medicine, and it has thus received the sanction of high scientific authority. The method consists in drawing teeth to the sounds of sweet music. A usually trying operation becomes, it is stated, not only painless, but quite pleasant, when conducted in this way. The physician addressed the Academy meeting on behalf of a Paris dentist who has already applied the musical method with success. The idea of the new process was first suggested by observation of patients under the influence of anesthetics, the drug used being nitrous oxid. As is known, the incipient effect of the latter is to produce in nearly every case disagreeable, sometimes almost unbearable, sensations, resembling those experienced in nightmare. The dentist in question came to the conclusion that this preliminary ordeal of bad dreams at the commencement of the anesthetic influence was caused by the perception of noises around by the mind when in a state of still partial consciousness. Why not soothe patients into the required condition of temporary oblivion by sweeter sounds? The dentist tried the experiment, and he and his patients were alike charmed with the result. The latter were as "good as gold," the tooth-pulling operations, not being interfered with by the groans or contortions of sufferers, were performed with the greatest ease, and on recovering consciousness all that the patients remembered were bars of the "Lohengrin" overture, for example, still softly singing in their ears. The new method having been invented nothing remained but to bring it to its greatest possible pitch of perfection. The obvious difficulties in the way of dentists habitually keeping bands, solo singers and choirs on the premises were avoided simply by means of powerful phonographs. Now the operator, whose new method has been revealed to the Academy of Medicine, regularly uses a machine of this kind. The patient puts the tubes to his ears, the music is started, the gas administered, and shortly afterward the occupant of the once-dreaded dental chair awakes from pleasant dreams to see the smiling surgeon showing him his tooth. Of course, a varied choice of musical selections can, and indeed should be, made to suit different temperaments and teeth. Without going into these particular questions, the physician spoke quite enthusiastically about the new process to the assembled academicians. He is eager to apply the musical method to surgical operations in general. He intends making experiments of his own in this direction, and he hoped that some of his hearers would do likewise.—*Dental Record.*

